# Chronic Vascular Complications in Diabetes

**DOI:** 10.1155/2013/858746

**Published:** 2013-06-26

**Authors:** Weiping Jia, Aimin Xu, Alex Chen, Jiarui Wu, Jianping Ye

**Affiliations:** ^1^Department of Endocrinology and Metabolism, Shanghai Diabetes Institute, Shanghai Jiao Tong University Affiliated Sixth People's Hospital, Shanghai 200233, China; ^2^The University of Hong Kong, Pokfulam, Hong Kong; ^3^University of Pittsburg, Pittsburgh, PA 15261, USA; ^4^Chinese Academy of Science, Shanghai 200031, China; ^5^Pennington Biomedical Research Center, Louisiana State University, Baton Rouge, LA 70808, USA

With high prevalence of type 2 diabetes worldwide, diabetic complications have been a major health problem, which reduce life quality and induce premature death in adults. The prevalence of type 2 diabetes is closely related to obesity and aging, two major risk factors for metabolic disorders. The diabetic complications, including retinopathy, nephropathy, neuropathy, skin ulcer, and atherosclerosis, are risk factors for mortality of patients. Retinopathy, nephropathy, and skin ulcer are representatives of microvascular complications, which often happen in insulin-deficient diabetic conditions, such as type 1 diabetic patients and some late-stage type 2 diabetic patients when *β*-cells completely lose function. Atherosclerosis that is often found in type 2 diabetes is a complication of macrovascular disease that increases the risk for myocardial infarction and strokes. However, atherosclerosis is receiving more and more appreciation in type 1 diabetes. Endothelial cells that line the inner vessel have been extensively studied in the mechanism of the diabetic complications. Impaired endothelial cell function is a common cellular mechanism for most of the diabetic complications (retinopathy, nephropathy, skin ulcer, and atherosclerosis). The cells are exposed to the high level of glucose in diabetic condition and subjected to glucose-induced stresses. The stress may cause overproliferation and deficiency in proliferation of endothelial cells, which contribute to dysfunction of microcirculation. Prevention and treatment of the complications are dependent on protection of the endothelial cell functions in most cases. In this issue, the mechanism of endothelial cell dysfunction was investigated in several studies to understand the molecular events by which the endothelial cells are impaired by hyperglycemia. This topic is enhanced with another group of studies about intervention of the endothelial complications with antioxidant compounds and new drug candidates. These studies will help readers to understand the current status and gain new insights into the diabetic complications.

*Weiping Jia* *Weiping Jia* *Aimin Xu* *Aimin Xu* *Alex Chen* *Alex Chen* *Jiarui Wu* *Jiarui Wu* *Jianping Ye* *Jianping Ye*

